# Impact of rapidly cleaving embryos on blastocyst formation and single-blastocyst transfer outcomes

**DOI:** 10.3389/fendo.2026.1745628

**Published:** 2026-02-18

**Authors:** Wen-jie Huo, Fei Peng, Chen Luo, Song Quan, Xiao-cong Wang

**Affiliations:** 1 Department of Obstetrics and Gynecology, Nanfang Hospital, Southern Medical University, Guangzhou, China; 2 Department of Psychology, School of Public Health, Southern Medical University, Guangzhou, China; 3 Department of Psychiatry, Zhujiang Hospital, Southern Medical University, Guangzhou, China

**Keywords:** blastocyst transfer, blastulation, embryo selection, *in vitro* fertilization, rapidly cleaving

## Abstract

**Background:**

Clarifying the impact of day-3 cell number on blastulation and subsequent pregnancy outcomes is essential for optimizing blastocyst selection criteria and refining embryo assessment protocols. While slow cleavage on day 3 is well-recognized as detrimental, the prognostic value of rapid cleavage (>8 cells) remains ambiguous.

**Methods:**

This retrospective cohort study (January 2015–April 2024) included 64,853 embryos undergoing blastocyst culture (Cohort 1) and 2,669 single-blastocyst frozen embryo transfer (FET) cycles (Cohort 2) at a large tertiary assisted reproduction center. Cohort 1 examined the association between day-3 cell number and blastulation potential. Cohort 2 evaluated clinical pregnancy, live birth, and miscarriage rates following single-blastocyst transfer using multivariable logistic regression adjusted for confounders. Embryos were stratified by maternal age (<35 or ≥35 years) and blastocyst grade (top-quality [≥4BB on day-5] or non-top-quality [≥3BC on day-5/6, excluding ≥4BB on day-5]).

**Results:**

In Cohort 1, compared to 8-cell embryos, 9- and 10-cell embryos had lower usable blastocyst rates (aORs [95% CI]: 0.77 [0.72–0.82] and 0.84 [0.77–0.91]); 11- and 12-cell embryos had comparable usable rates (0.96 [0.85–1.09] and 1.08 [0.93–1.24]) but higher top-quality rates (1.59 [1.37–1.85] and 2.17 [1.85–2.54]); and embryos with ≥13 cells had higher rates for both usable and top-quality blastocysts (all aORs > 1.4; 95% CIs excluded 1). This pattern was consistent across female age subgroups. In Cohort 2, however, the advantage of 11–16-cell embryos translated into superior pregnancy and live birth rates only in younger women receiving top-quality blastocysts versus 8-cell embryos (76.5% *vs*. 63.0%, P = 0.002, aOR = 1.95 [1.30–2.96]; 61.2% *vs*. 51.2%, P = 0.034, aOR = 1.58 [1.10–2.30]). Conversely, in older women with non-top-quality blastocysts, 11–16-cell embryos predicted lower pregnancy and live birth rates (26.5% *vs*. 51.0%, P = 0.023, aOR = 0.40 [0.15–0.97]; 14.7% *vs*. 38.5%, P = 0.019, aOR = 0.32 [0.10–0.89]). The 9–10-cell embryos generally showed outcomes comparable to 8-cell embryos, except for a reduced live birth rate in the older, non-top-quality blastocyst subgroup (23.9% *vs*. 38.5%, P = 0.047, aOR = 0.51 [0.26–0.98]).

**Conclusion:**

Day-3 cell number serves as a context-dependent prognostic indicator for optimizing blastocyst selection. For young women with top-quality blastocysts, ≥11-cell embryos are the strongest candidates; conversely, 8-cell embryos appear optimal for older women with non-top-quality blastocysts.

## Introduction

Blastocyst transfer on day 5 or 6 has become a cornerstone of modern *in vitro* fertilization and embryo transfer (IVF-ET), as it generally achieves higher implantation rates than cleavage-stage (day-3) transfers (1). In these cycles, elective single embryo transfer is considered the preferable approach towards safe and effective IVF (2). However, blastocyst selection methods based mainly on blastocyst formation timing and morphology offer only limited predictive value for pregnancy outcomes (3–5). Consequently, there is a growing emphasis on identifying additional markers of blastocyst viability (5).

Several alternative predictors have been investigated, including time-lapse parameters, chromosomal status, and other morphological features (6–10). Yet time-lapse monitoring and genetic screening are not universally accessible. As a result, additional morphology remains the most widely available and cost-effective marker of developmental potential. Recent evidence indicates that day-3 cell number might influence blastocyst transfer outcomes. Specifically, slow-cleaving embryos (≤5/≤6 cells) are associated with lower blastocyst live birth rates compared to normal 8-cell embryos (9–11). However, the impact of rapid cleavage is controversial, with outcomes varying between comparable and superior to those of 8-cell embryos, necessitating further study.

A critical limitation in understanding fast-cleaving embryos is the lack of detailed stratification analysis. Since a critical threshold (10–12 cells) exists within the 9–16 cell range, beyond which embryos demonstrate significantly lower aneuploidy rates and fewer cleavage anomalies (12–14), grouping all rapidly cleaving embryos into such a broad category obscures their distinct developmental potentials (10, 11, 15). Besides, the influence of rapid cleavage maybe heterogeneous across different blastocyst quality and maternal age subgroups. Existing evidence comes predominantly from young populations (9, 16, 17), limiting generalizability in the context of steadily increasing maternal age (18, 19). To address these critical gaps, systematical subgroup analyses that categorize rapid cleavage by specific cell count are urgently needed.

Equally important is whether embryos reach the blastocyst stage in the first place, since blastocyst availability is a prerequisite for transfer. Although extended culture is widely practiced, its non-selective application carries a higher risk of cycle cancellation (20–22). This has motivated ongoing efforts to improve the identification of embryos with blastulation competence (23, 24). Recent evidence suggests that rapidly cleaving embryos can achieve equal or better blastulation outcomes compared with the traditional 8-cell benchmark, challenging long-held assumptions (14, 25, 26). Therefore, evaluating how rapid cleavage influences both blastulation and subsequent pregnancy outcomes is critical for refining embryo selection strategies.

This study aimed to systematically evaluate the impact of rapid cleavage on both blastulation and subsequent pregnancy outcomes. By benchmarking against the conventional 8-cell stage and considering maternal age, blastocyst quality, and detailed day-3 cell count categories, we sought to clarify the clinical contexts in which rapid cleavage may serve as a prognostic indicator. Such evidence could contribute to refining blastocyst selection criteria and advancing more personalized approaches to IVF practice.

## Materials and methods

### Participants and dataset

This retrospective cohort study included patients who underwent IVF or intracytoplasmic sperm injection (ICSI) treatment at the Center for Reproductive Medicine, Nanfang Hospital, Southern Medical University, China, between January 2015 and April 2024. This study was reviewed and approved by the Institutional Review Board of Nanfang Hospital (Ethical approval number: NFEC-2025-452) and conducted in accordance with the Declaration of Helsinki. Informed consent was waived because all data used here were fully anonymized.

We analyzed two cohorts. Cohort 1 (Blastulation analyses) included all embryos cultured to the blastocyst stage with complete parameter records. After excluding embryos with missing annotations or day-3 counts outside 4–16-cell or day-2 counts outside 2–6-cell, 64, 853 embryos from 12, 474 IVF/ICSI cycles were included in the analysis. Cohort 2 (Blastocyst transfer analyses) included all single day-5 or day-6 blastocyst transfers in frozen-thawed embryo transfer (FET) cycles. After the same exclusions, 2,669 embryos were included in the analysis. These were further stratified by maternal age at oocyte pick-up and blastocyst quality: (1) young maternal age (<35 years), top-quality blastocyst transfer (n = 1,098); (2) young maternal age (<35 years), non-top-quality blastocyst transfer (n = 830); (3) advanced maternal age (≥35 years), top-quality blastocyst transfer (n = 327); (4) advanced maternal age (≥35 years), non-top-quality blastocyst transfer (n = 414). Day-3 cell number were categorized into four groups: 4–7-cell, 8-cell(reference), 9–10-cell, and 11–16-cell.

### Ovarian stimulation and embryo culture

Ovarian stimulation was performed using standard GnRH-agonist or GnRH-antagonist protocols. A subset of patients received alternative stimulation approaches, including minimal stimulation, progestin-primed ovarian stimulation and luteal phase ovarian stimulation. Final oocyte maturation was triggered when at least one follicle reached 18 mm, using 2000–10000 IU hCG (Livzen, China) or 250 μg Ovidrel (Merck-Serono, Switzerland), with or without 0.2 mg Triptorelin (Decapeptyl, Ferring, Switzerland). Oocyte retrieval was performed 34–36 hours later under transvaginal ultrasound guidance. Fertilization was achieved by conventional IVF or ICSI; in severe male-factor cases, sperm retrieval was performed via percutaneous epididymal sperm aspiration (PESA) or testicular sperm aspiration (TESA). After insemination, embryos were cultured in pre-equilibrated cleavage medium under mineral oil at 37 °C, 6% CO_2_, and 5% O_2_. At 68 ± 2 hours post insemination (day-3), blastomere count, fragmentation, and symmetry were assessed according to Istanbul consensus guidelines (27). Selected embryos were cultured further in blastocyst medium to day-5 or day-6 and were then graded according to Gardner criteria (expansion graded 1-6; the inner cell mass and trophectoderm graded A-C).

### Endometrial preparation protocols

Endometrial preparation was conducted following three protocols: 1) Natural cycle: ultrasonographic monitoring was initiated on menstrual days 10–12. When the leading follicle reached 14 mm, urinary LH was assessed three times daily using test strips. Upon observation of a qualitative color change (from weak to positive), serum LH testing was performed via electrochemiluminescence immunoassay. After detection of the LH surge (by serum testing) and an endometrial thickness ≥8 mm, luteal support was initiated with either vaginal or oral progesterone on the following day (designated as progesterone day-0). Embryo transfer was performed on progesterone day-5. 2) Hormone replacement therapy (HRT) cycle: oral estradiol valerate was initiated on menstrual days 2–4 or after full pituitary suppression was achieved with a GnRH agonist, either as a fixed dose (6 mg/day) or in a step-up regimen (4→6→8 mg/day) for 10–12 days. If endometrial thickness did not reach ≥8 mm, the dose was increased to 10 mg/day until this threshold was met. Then luteal support was initiated (designated as progesterone day-0). Embryo transfer was performed on progesterone day-5. 3) Ovulation induction (OI): Letrozole (2.5–5.0 mg/day for 5 days) was initiated on menstrual cycle days 2–5, either alone or supplemented with human menopausal gonadotropin (HMG; 37.5–75 IU/day). Luteal support was started on the day after the detection of the LH surge (designated progesterone day 0). Embryo transfers were performed on progesterone day 5.

### Feature collection

For the blastulation analyses (cohort 1), primary predictors comprised day-3 blastomere counts (4–16). Baseline and other embryo characteristics were collected, including female age, infertility type (primary or secondary), stimulation protocol (agonist, antagonist, or unconventional), the number of oocytes retrieved, fertilization method (IVF or ICSI), pronuclear (PN) pattern (2PN or non-2PN), day-2 cell number, day-3 fragmentation rate (<10% or ≥10%), symmetry (symmetric or asymmetric), day-3 observation time recorded as hours post insemination (hpi), and patient identifier. Two primary blastulation outcomes were evaluated: usable blastocyst (blastulation) was defined as ≥3BC on day-5 or day-6; top-quality blastocyst was defined as ≥4BB on day-5.

For the blastocyst transfer analysis (cohort 2), primary predictors comprised day-3 blastomere counts (4–7, 8, 9–10 or 11–16). Potential confounders included blastocyst morphology (expansion: 3–6; inner cell mass: A/B; trophectoderm: A/B/C), day of blastocyst formation (5 or 6), and cycle factors (female age, endometrial thickness on transfer day and endometrial preparation protocol [natural cycle, hormone replacement, hormone replacement + gonadotropin-releasing hormone agonist, or ovulation induction]). Three primary pregnancy outcomes were evaluated: the clinical pregnancy rate (CPR) was defined as the number of transfer cycles resulting in detection of both a gestational sac and fetal heartbeat at 4 weeks post–blastocyst transfer, divided by the total number of embryo transfer cycles. The miscarriage rate was calculated as the number of miscarriages divided by the number of clinical pregnancies. The live birth rate (LBR) was calculated as the number of live births divided by the total number of embryo transfer cycles.

### Statistical analysis

Continuous variables are presented as mean ± standard deviation (SD), while categorical variables are presented as percentages. Differences in baseline characteristics (including potential confounders) and outcomes between the cell number groups were assessed using t-tests for continuous variables and χ² tests for categorical variables. Statistical significance for these unadjusted group comparisons was defined as a two-tailed p-value < 0.05.

Multivariate logistic regression analyses were conducted to assess the independent association between day−3 cell number and each outcome (two blastulation and three pregnancy outcomes). Confounders for each model were selected via a best−subset approach by comparing all possible variable combinations and retaining the model with the minimum Akaike Information Criterion (AIC) value. For blastulation outcomes (involving multiple embryos per cycle), generalized linear mixed−effects models (GLMMs) were employed, incorporating random intercepts for patient identifier and day-3 observation time. For blastocyst transfer outcomes, generalized estimating equations (GEE) logistic regression was used. All models were adjusted for their corresponding confounders, and results are reported as adjusted odds ratios (aORs) with 95% confidence intervals (CIs).

## Results

### Comparison of blastulation outcomes

**Figure 1:**
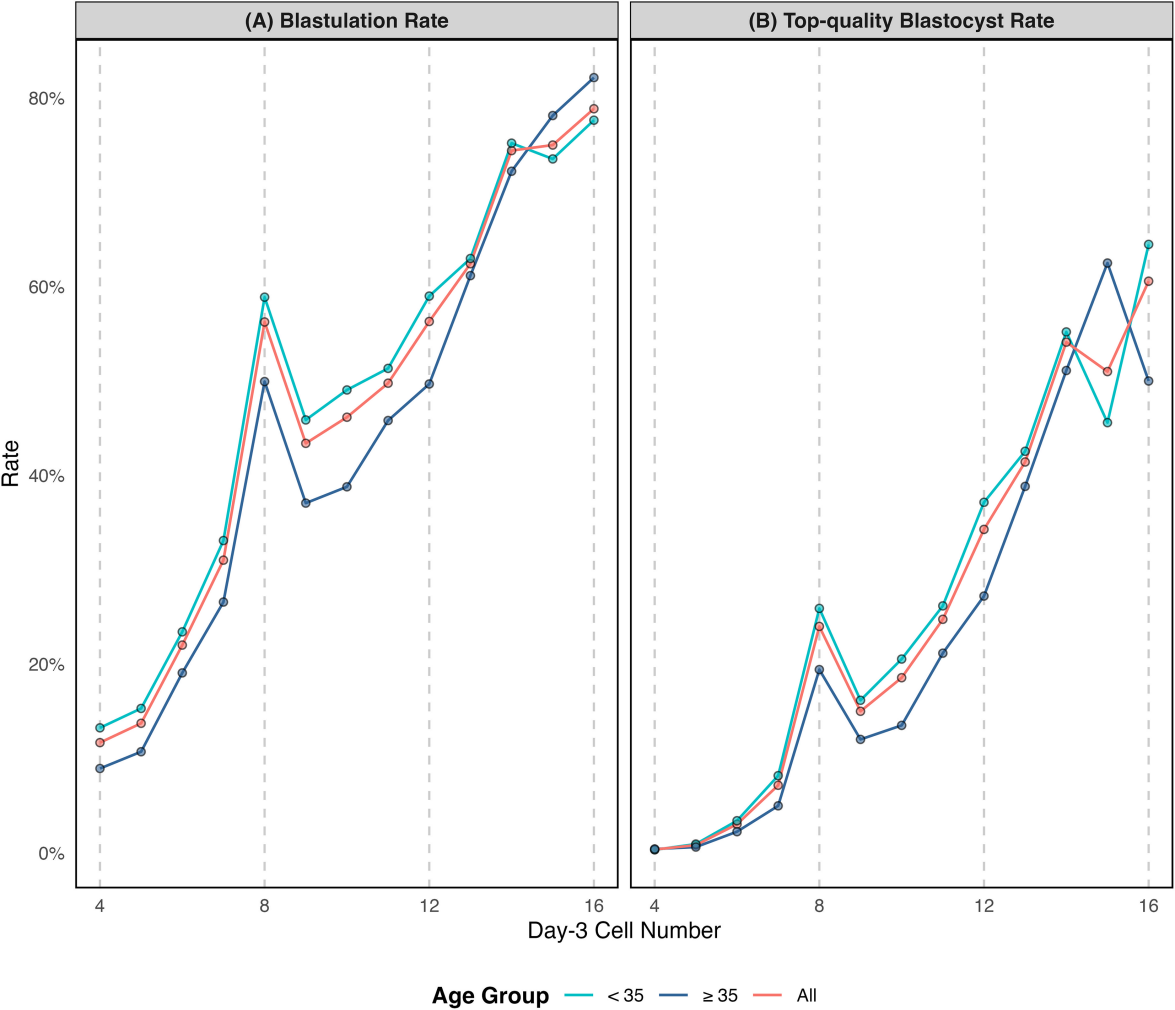
Association between day-3 cell count and blastocyst formation, stratified by maternal age. **(A)** Blastulation rate (number of usable blastocysts formed per day-3 embryo). **(B)** Top-quality blastocyst rate (number of top-quality blastocysts per day-3 embryo).

A total of 64,853 day-3 embryos from Cohort 1 underwent extended culture to day-5/6. Among these, 21,749 (33.5%) developed into usable blastocysts, and 7,221 (11.1%) were top-quality blastocysts (≥4BB on day 5). Baseline cycle characteristics and other embryo parameters are detailed in **Table 1**. Both blastulation rates and top-quality blastocyst rates, as well as their corresponding aORs across day-3 cell number groups, are presented in **Figure 1** and **Table 2**.

**Table 1:** Baseline characteristics of embryos undergoing extended culture.

Characteristics	Day-3 cell number			
	4-7 (n=36693)	8 (n=12188)	9-10 (n=12481)	11-16 (n=3491)
Female age	32.5 ± 4.9	32.0 ± 4.6	31.8 ± 4.8	31.8 ± 4.8
Infertility type (Primary)	39.6%	37.7%	41.5%	42.9%
Stimulation protocol				
Agonist	50.6%	53.0%	51.9%	52.0%
Antagonist	43.6%	43.3%	43.7%	43.2%
Unconventional	5.8%	3.7%	4.4%	4.8%
Number of oocytes retrieved	16.2 ± 8.5	18 ± 8.4	17.1 ± 8.5	17.9 ± 8.4
Fertilization method (IVF)	76.3%	79.8%	75.4%	72.8%
Pronuclear (2PN)	87.6%	83.5%	89.3%	84.9%
Day-2 cell number	3.5 ± 1.2	4.2 ± 1.2	4.9 ± 1.2	5.3 ± 1.2
Day-3 fragmentation rate (<10%)	36.6%	58.2%	48.8%	75.9%
Day-3 symmetry	73.4%	83.1%	85.3%	96.6%
Day-3 observation time(hpi)	67.0 ± 0.6	67.1 ± 0.7	67.0 ± 0.6	67.0 ± 0.6

IVF, *in vitro* fertilization; hpi, hours after insemination.

**Table 2:** Blastulation outcomes across day-3 cell number.

Day-3 cell number	Sample	Blastulation		Top-quality blastocyst	
		Rate	aOR(95%CI)	Rate	aOR(95%CI)
4	7188	0.117	0.11 (0.10–0.12)	0.004	0.02 (0.02–0.04)
5	8912	0.137	0.15 (0.14–0.16)	0.008	0.05 (0.04–0.07)
6	12111	0.22	0.26 (0.24–0.28)	0.03	0.15 (0.14–0.18)
7	8482	0.31	0.49 (0.46–0.53)	0.072	0.38 (0.35–0.43)
8	12188	0.563	reference	0.24	reference
9	8010	0.434	0.77 (0.72–0.82)	0.15	0.86 (0.79–0.95)
10	4471	0.462	0.84 (0.77–0.91)	0.186	1.13 (1.02–1.26)
11	1515	0.498	0.96 (0.85–1.09)	0.248	1.59 (1.37–1.85)
12	1094	0.563	1.08 (0.93–1.24)	0.343	2.17 (1.85–2.54)
13	338	0.624	1.42 (1.11–1.83)	0.414	3.06 (2.36–3.97)
14	340	0.744	2.54 (1.92–3.34)	0.541	4.62 (3.57–5.96)
15	100	0.75	3.19 (1.88–5.41)	0.51	3.92 (2.50–6.16)
16	104	0.788	2.98 (1.77-5.02)	0.606	6.97 (4.38–11.10)

aOR, adjusted odds ratio; CI: confidence interval.

aORs were derived from mixed-effects logistic regression models, adjusted for female age, stimulation protocol, fertilization method, pronuclear count, day-2 cell number, day-3 fragmentation rate, and day-3 symmetry, with random intercepts for day-3 observation time and patient identification code. The model for top-quality blastocyst additionally adjusted for infertility type.

Compared to 8-cell embryos (rate: 56.3%), usable blastocyst rates were significantly lower in the 4-, 5-, 6-, 7-, 9- and 10-cell groups (rates: 11.7–46.2%; aORs: 0.11–0.84, all 95% CIs excluded 1), higher in the 13-, 14-, 15- and 16-cell groups (rates: 62.4–78.8%; aORs: 1.42–3.19, all 95% CIs excluded 1), and comparable in the 11- and 12-cell groups (rates: 49.8% and 56.3%; aORs: 0.96 [0.85–1.09] and 1.08 [0.93–1.24]). A similar pattern was observed for top-quality blastocysts, but the positive shift was from an earlier cell-number threshold: relative to the 8-cell (rate: 24.0%), the top-quality blastocyst rate was significantly lower in the 4-, 5-, 6-, 7- and 9-cell groups group (rates: 0.4%–15.0%; aORs: 0.02–0.86, all 95% CIs excluded 1), but higher from the 10-cell group onward (rates:18.6%–60.6%; aORs:1.13–6.97, all 95% CIs excluded 1).

Subgroup analyses stratified by maternal age (<35 or ≥35 years) generally mirrored the overall cohort trends (**Figure 1** and **STable 1**). Exceptions were noted in the <35 years subgroup, where 13-cell embryos exhibited comparable usable rates to 8-cell controls (aOR: 1.30 [0.96–1.75]). In the ≥35 years subgroup, 9- and 10-cell embryos had a comparable top-quality rate to 8-cell controls (aORs: 0.87 [0.73–1.04] and 1.01 [0.81–1.25]).

### Comparison of main pregnancy outcomes and subgroup analysis

Cohort 2 included 2,669 single frozen-thawed blastocyst transfer cycles. The overall CPR was 55.3%, LBR was 42.1%, and miscarriage rate was 24.0%. The mean maternal age at oocyte retrieval was 32 years and 33 years at embryo transfer. The sample sizes and number of top-quality blastocysts per day-3 cell number group were as follows: 4–7-cell (n = 829; 236), 8-cell (n = 866; 573), 9–10-cell (n = 669; 389), and 11–16-cell (n = 305; 227). The proportion of top-quality blastocysts was significantly higher in the 11–16-cell group than in the 8-cell group (74.4% *vs*. 66.2%, P = 0.008), and significantly lower in the 4–7, and 9–10-cell groups (28.5%, 58.1% *vs*. 66.2%, all P < 0.001). Baseline characteristics differed significantly among day-3 cell number groups (**Table 3**) and were adjusted as covariates in subsequent logistic regression analyses.

**Table 3:** Characteristics of embryos undergoing frozen-thawed single blastocyst transfer across day-3 cell number.

Characteristics	Day-3 cell number				
	4-7 (n=829)	8 (n=866)	9-10 (n=669)	11-16 (n=305)	P-value
Female age (OPU)	32.2 ± 4.4	31.8 ± 4.4	31.7 ± 4.4	32.2 ± 4.4	0.048
Female age (ET)	33.3 ± 4.4	32.6 ± 4.4	32.9 ± 4.4	33.2 ± 4.4	0.003
Endometrium thickness (mm)	9.4 ± 2.1	9.3 ± 2.1	9.4 ± 2.1	9.4 ± 2.1	0.806
Endometrial preparation (%)					0.018
HRT cycle	67.10%	67.90%	68.30%	77.40%	
HRT+GnRH-a cycle	12.50%	15.20%	13.50%	11.50%	
Natural cycle	13.40%	11.80%	12.60%	8.50%	
Ovarian induction cycle	7%	5.10%	5.70%	2.60%	
Day of blastocyst formation					<0.001
5	42.30%	82.40%	74%	85.90%	
6	57.70%	17.60%	26%	14.10%	
Expansion grade					<0.001
3	19.20%	15.10%	13.60%	7.50%	
4	71.20%	79.70%	77.40%	84.30%	
5	6.60%	3.60%	4.80%	4.90%	
6	3%	1.60%	4.20%	3.30%	
ICM grade					<0.001
A	10%	22.70%	18.80%	30.20%	
B	90%	77.30%	81.20%	69.80%	
TE grade					<0.001
A	11.70%	21.90%	18.40%	31.80%	
B	75.40%	72.20%	72.20%	58.70%	
C	12.90%	5.90%	9.40%	9.50%	

OPU, oocyte pick-up; ET, embryo transfer; HRT, hormone replacement treatment; GnRH-a, gonadotropin-releasing hormone agonist; ICM, inner cell mass; TE, trophectoderm.

In the overall cohort2, neither the 9–10-cell nor 11–16-cell groups differed significantly from the 8-cell group in CPR (57.1% and 63.3% *vs*. 57.9%, P = 0.808 and 0.112, aOR = 1.05[0.85-1.30] and 1.24 [0.94-1.64]) or LBR (43.6% and 46.6% *vs*. 44.8%, P = 0.689 and 0.644, aOR = 1.03 [0.83-1.27] and 1.09 [0.83-1.44]). The 4–7-cell group showed significantly lower CPR (48.5% *vs*. 57.9%, P < 0.001) and LBR (36.3% *vs*. 44.8%, P < 0.001); however, these differences were not significant after adjustment to confounders (see **Table 4**).

**Table 4:** Logistic regression analysis of blastocyst transfer outcomes across day-3 cell number.

Outcomes	Day-3 cell number (aOR [95%CI])			
	4-7	8	9-10	11–16
Overall (n=2669)				
Pregnancy	0.96 (0.78–1.19)	reference	1.05 (0.85–1.30)	1.24 (0.94–1.64)
Live birth	0.96 (0.77–1.19)	reference	1.03 (0.83–1.27)	1.09 (0.83–1.44)
Miscarriage	1.09 (0.78–1.52)	reference	1.06 (0.77–1.48)	1.19 (0.79–1.76)
Age<35&Top-quality blastocyst (n=1098)				
Pregnancy	1.15 (0.80–1.67)	reference	1.33 (0.97–1.82)	1.95 (1.30–2.96)
Live birth	1.18 (0.84–1.69)	reference	1.12 (0.84–1.51)	1.58 (1.10–2.30)
Miscarriage	0.85 (0.47–1.49)	reference	1.22 (0.78–1.92)	0.99 (0.57–1.69)
Age<35&Non-top-quality blastocyst (n=830)				
Pregnancy	0.89 (0.62–1.29)	reference	0.85 (0.56–1.28)	0.49 (0.24–0.98)
Live birth	0.95 (0.66–1.39)	reference	0.99 (0.65–1.52)	0.67 (0.31–1.39)
Miscarriage	0.93 (0.51–1.70)	reference	0.70 (0.34–1.39)	0.58 (0.12–2.08)
Age≥35&Top-quality blastocyst (n=327)				
Pregnancy	0.73 (0.37–1.42)	reference	1.01 (0.58–1.77)	1.91 (0.96–3.92)
Live birth	0.61 (0.28–1.28)	reference	1.29 (0.72–2.30)	1.05 (0.52–2.09)
Miscarriage	1.90 (0.67–5.42)	reference	0.63 (0.25–1.54)	1.67 (0.70–4.03)
Age≥35&Non-top-quality blastocyst (n=414)				
Pregnancy	0.58 (0.34–0.99)	reference	0.71 (0.38–1.30)	0.40 (0.15–0.97)
Live birth	0.50 (0.28–0.87)	reference	0.51 (0.26–0.98)	0.32 (0.10–0.89)
Miscarriage	1.69 (0.69–4.27)	reference	2.28 (0.83–6.44)	2.23 (0.43–11.16)

aOR, adjusted odds ratio; CI, confidence interval.

Adjusted for female age, endometrium thickness, endometrial preparation protocol, day of blastocyst formation, expansion grade, ICM grade and TE grade.

**Figure 2:**
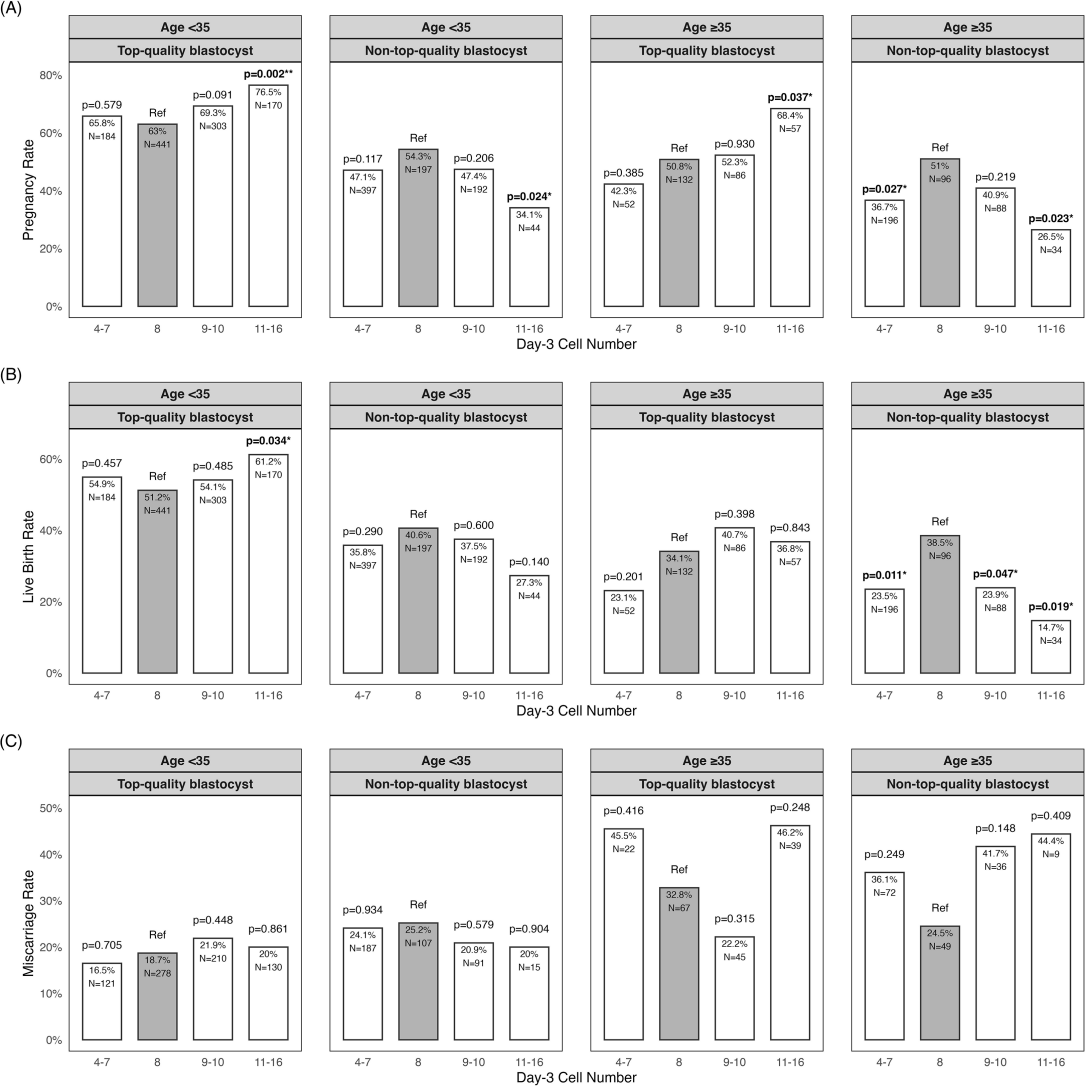
Main pregnancy outcomes by day-3 cell count, stratified by maternal age and blastocyst quality. **(A)** Pregnancy rate (number of clinical pregnancies per embryo transfer). **(B)** Live birth rate (number of live births per embryo transfer). **(C)** Miscarriage rate (number of miscarriages per clinical pregnancy). p<0.05, *p<0.01.

Subgroup analyses revealed that blastocyst quality and maternal age significantly modified the association between day-3 cell number and pregnancy outcomes (**Figure 2** and **Table 4**). Among women <35 years receiving top-quality blastocysts, 11–16-cell embryos were associated with significantly higher CPR (76.5% *vs*. 63.0%; P = 0.002; aOR = 1.95 [1.30–2.96]) and LBR (61.2% *vs*. 51.2%; P = 0.034; aOR = 1.58 [1.10–2.30]) compared to 8-cell embryos, with comparable miscarriage rates. Both 9–10-cell and 4–7-cell embryos showed outcomes similar to 8-cell embryos across all three endpoints.

Conversely, among women ≥35 years receiving non-top-quality blastocysts, both 11–16-cell and 4–7-cell embryos demonstrated significantly lower CPR (26.5% and 36.7% *vs*. 51.0%; P = 0.023 and 0.027; aOR = 0.40 [0.15–0.97] and 0.58 [0.34–0.99]) and LBR (14.7% and 23.5% *vs*. 38.5%; P = 0.019 and 0.011; aOR = 0.32 [0.10–0.89] and 0.50 [0.28–0.87]) versus 8-cell embryos, with no difference in miscarriage rates. The 9–10-cell group showed comparable CPR and miscarriage rates but a significantly reduced LBR (23.9% *vs*. 38.5%; P = 0.047; aOR = 0.51 [0.26–0.98]).

In the remaining two subgroups (women <35 years with non-top-quality blastocysts and ≥35 years with top-quality blastocysts), the link between day-3 cell number and blastocyst transfer outcomes was minimal. Although younger women with non-top-quality blastocysts showed a reduction in CPR with 11–16-cell embryos (34.1% *vs* 54.3%; P = 0.024; aOR = 0.49 [0.24–0.98]), this did not translate into a significant difference in LBR or miscarriage outcomes. All other comparisons in these subgroups showed no difference in outcomes relative to the 8-cell reference.

## Discussion

This study utilized two cohorts to systematically evaluate the relationship between day-3 rapid cleavage and subsequent developmental competence, including blastulation potential and post-blastulation transfer outcomes. We found that rapid cleavage represented a context-dependent phenomenon: one detrimental and one beneficial, depending on maternal age, blastocyst quality, and specific cell count. These results refine the clinical interpretation of rapid cleavage and underscore the importance of early morphological parameters in blastocyst transfer cycles.

Our results suggest that the blastulation competence of rapidly cleaving embryos was superior or inferior to that of 8-cell embryos depending on whether they exceeded the 10–12-cell threshold. This model is in line with previous findings that aneuploidy and abnormal cleavage patterns in >8-cell embryos are mostly confined to the lower cell number range (e.g., 9–10 cells; 12–14, 25, 28). Given that our static morphology evaluation could not capture dynamic abnormalities such as direct cleavage, they remain a potential confounder in interpreting our results. Nevertheless, the nuanced links we observed between day-3 rapid cleavage (>8 cells) and blastulation as well as subsequent live birth offer practical framework, especially for conventional static observation systems. Collectively, our large cohort1 reveal a dual-directional relationship between day-3 cell number and blastulation outcomes, challenging the historical view of rapid cleavage as a uniformly negative indicator.

The central finding identifies a specific favorable scenario: in young patients receiving top-quality blastocysts, 11–16-cell embryos demonstrated superior live birth rates compared to 8-cell embryos. This observation echoes a recent study, which found higher LBR from >9-cell embryos in younger patients receiving day-5 blastocyst (16). Younger maternal age and high blastocyst quality are established markers of euploid status (29–32). Our data further indicate that their combination constitutes a necessary precondition for the superiority of 11–16-cell embryos, a conclusion supported by the reversed trend observed with low-quality blastocysts. Furthermore, transcriptomic evidence indicates that developmental speed reflects underlying molecular states and that its biological meaning may be modified by maternal age (33). This understanding therefore supports that day-3 11–16-cell embryos may be a positive indicator, but only when multiple top-quality blastocysts are available from young patients.

Conversely, among older patients receiving non-top-quality blastocysts, both 9–10-cell and 11–16-cell were related to lower LBR compared to 8-cell. This reversal may operate through distinct mechanisms: rapid cleavage in younger oocytes reflects robust cell−cycle and metabolic coordination, whereas in older oocytes, it frequently results from mitotic instability combined with diminished DNA repair capacity (32–34). This compromised state is often linked to an elevated incidence of chromosomal abnormalities (31, 32). Although such embryos can develop into blastocysts, underlying biological defects likely contribute to pregnancy failure (35, 36). Notably, this negative effect has not been widely reported, as previous studies primarily focus on indicated neutral or beneficial outcomes (10, 11). The discrepancy might be due to an insufficient sample size in this subgroup for robust statistical analysis. Collectively, our novel finding indicates that when multiple non-top-quality blastocysts are available from older patients, those derived from 8-cell embryos are superior to those from 9–16-cell.

Based on our findings, day-3 cleavage speed retains important prognostic value for developmental outcomes even after blastulation. This clinical relevance may be grounded in developmental biology, as day 3 coincides with the major wave of zygotic genome activation (37). Rather than a simple measure of timing, cleavage at this stage likely reflects fundamental qualitative differences in the maternal-to-zygotic transition, resulting from either efficient cell cycle coordination, dysregulated division prior to complete genomic activation, or compensatory acceleration due to underlying deficiencies (38–40). Therefore, day-3 rapid cleavage is better interpreted as a context-dependent prognostic indicator, rather than an independent biomarker.

A major strength of this study is its detailed subgroup stratification based on precise day-3 cell count categories. In contrast, earlier literature often aggregated all >8-cell embryos into a single group or failed to adjust for critical effect modifiers such as age and blastocyst quality (10, 11, 13, 26, 41). By accounting for these confounders, our approach helps resolve inconsistencies in previous studies where the masking of opposing effects may have obscured true associations. Furthermore, day-3 cell number was identified as a context-dependent indicator for blastocyst selection rather than an inherently detrimental or beneficial feature.

Several study limitations merit consideration. First, while outcomes strongly implicate age-mediated aneuploidy, definitive confirmation via preimplantation genetic testing (PGT) for aneuploidies validation is needed. Second, our static morphological assessment cannot identify abnormal cleavage like direct cleavage, which might be an unmeasured biological confounder. Future time-lapse studies controlling for such abnormalities are warranted. Third, larger multicenter cohorts focusing on rapid-cleaving embryos’ effect are required to consolidate the observed advantage and disadvantage.

## Conclusion

In conclusion, this study demonstrates that the prognostic value of day-3 rapid cleavage is highly context dependent. Notably, only embryos with 11–16 cells from younger women consistently outperformed the conventional 8-cell benchmark, whereas rapid cleavage was detrimental in older patients with non-top-quality blastocysts. These findings challenge traditional embryo assessment paradigms and underscore the continued clinical relevance of precise early-stage morphological evaluation. Incorporating such stratified criteria may improve blastocyst selection strategies and support more personalized approaches to IVF.

## Data Availability

The raw data supporting the conclusions of this article will be made available by the authors, without undue reservation.
