# Supplementary material for: Impact of rapidly cleaving embryos on blastocyst formation and single-blastocyst transfer outcomes
Source: Front Endocrinol (Lausanne). 2026 Feb 18;17:1745628. doi: 10.3389/fendo.2026.1745628 (PMC12956653; doi:10.3389/fendo.2026.1745628)
Supplement: Supplementary file 1 [file Table1.docx]

**STable1.** Sub-group analysis of blastulation outcomes by day-3 cell number, stratified by female age.

| Day-3 cell number | Female age< 35 | | | Female age≥ 35 | | |
| --- | --- | --- | --- | --- | --- | --- |
|  | Sample | Blastulation  aOR(95%CI) | Top-quality blastocyst  aOR(95%CI) | Sample | Blastulation  aOR(95%CI) | Top-quality blastocyst  aOR(95%CI) |
| 4 | 4572 | 0.11 (0.10-0.12) | 0.02 (0.01-0.03) | 2616 | 0.11 (0.09-0.13) | 0.03 (0.02-0.07) |
| 5 | 5853 | 0.15 (0.14-0.17) | 0.05 (0.04-0.07) | 3059 | 0.15 (0.13-0.18) | 0.05 (0.03-0.08) |
| 6 | 8205 | 0.25 (0.23-0.27) | 0.16 (0.14-0.19) | 3906 | 0.30 (0.26-0.33) | 0.14 (0.11-0.19) |
| 7 | 5777 | 0.49 (0.45-0.53) | 0.40 (0.35-0.45) | 2705 | 0.53 (0.46-0.60) | 0.33 (0.26-0.41) |
| 8 | 8600 | reference | reference | 3588 | reference | reference |
| 9 | 5757 | 0.77 (0.71-0.83) | 0.85 (0.77-0.94) | 2253 | 0.78 (0.69-0.89) | 0.87 (0.73-1.04) |
| 10 | 3213 | 0.85 (0.77-0.93) | 1.17 (1.04-1.32) | 1258 | 0.85 (0.73-0.98) | 1.01 (0.81-1.25) |
| 11 | 1085 | 0.91 (0.79-1.05) | 1.58 (1.33-1.88) | 430 | 1.07 (0.85-1.35) | 1.60 (1.20-2.15) |
| 12 | 778 | 1.09 (0.92-1.29) | 2.18 (1.81-2.61) | 316 | 1.12 (0.86-1.45) | 2.05 (1.50-2.79) |
| 13 | 235 | 1.30 (0.96-1.75) | 2.58 (1.89-3.52) | 103 | 2.15 (1.37-3.39) | 3.98 (2.48-6.39) |
| 14 | 250 | 2.39 (1.72-3.30) | 4.58 (3.40-6.17) | 90 | 2.88 (1.72-4.82) | 4.91 (2.99-8.08) |
| 15 | 68 | 2.27 (1.23-4.19) | 2.83 (1.66-4.85) | 32 | 5.29 (2.04-13.70) | 11.10 (4.71-26.20) |
| 16 | 76 | 2.65 (1.43-4.92) | 7.00 (4.03-12.10) | 28 | 5.64 (1.98-16.10) | 6.83 (2.90-16.10) |

Abbreviations: aOR, adjusted odds ratio; CI: confidence interval.

aORs were derived from mixed-effects logistic regression models, adjusted for female age, stimulation protocol, fertilization method, pronuclear pattern, day-2 cell number, day-3 fragmentation rate, and day-3 symmetry, with random intercepts for day-3 observation time and patient identification code. The model for top-quality blastocyst additionally adjusted for infertility type.
